# Explaining discrepancies in self-reported quality of life in frail older people: a mixed-methods study

**DOI:** 10.1186/s12877-017-0641-y

**Published:** 2017-10-26

**Authors:** Anne van der Vorst, G. A. Rixt Zijlstra, Nico De Witte, Ruth G. M. Vogel, Jos M. G. A. Schols, Gertrudis I. J. M. Kempen, D-SCOPE Consortium, Peter Paul De Deyn, Liesbeth De Donder, Jan De Lepeleire, Ellen De Roeck, Nico De Witte, Eva Dierckx, Daan Duppen, Sarah Dury, Sebastiaan Engelborghs, Bram Fret, Lieve Hoeyberghs, Tinie Kardol, Gertrudis I. J. M. Kempen, Deborah Lambotte, Birgitte Schoenmakers, Jos M. G. A. Schols, An-Sofie Smetcoren, Michaël Van Der Elst, Anne van der Vorst, Dominique Verté, G. A. Rixt Zijlstra

**Affiliations:** 1 Department of Health Services Research, Care and Public Health Research Institute (CAPHRI), Maastricht University, P.O. Box 616, 6200 MD Maastricht, the Netherlands; 2 Faculty of Psychology and Educational Sciences, Vrije Universiteit Brussel, Brussels, Belgium; 3 Faculty of Education, Health and Social Work, University College Ghent, Ghent, Belgium; 4 Research Centre for Community Care, Zuyd Hogeschool, Heerlen, the Netherlands; 5 Department of Family Medicine, Care and Public Health Research Institute (CAPHRI), Maastricht University, Maastricht, the Netherlands

**Keywords:** Community-dwelling, Multidimensional frailty, Well-being; successful aging, Strengths-based approach

## Abstract

**Background:**

Most research on multidimensional frailty focuses on deficits and risks of adverse outcomes. However, although some frail older people report a low quality of life (QoL), others still report a relatively high QoL. More knowledge about these discrepancies might give new insight into developing frailty prevention strategies. Therefore, this mixed-method study aimed (a) to identify characteristics related to QoL among frail older people; and (b) to explain discrepancies between higher and lower levels of QoL, with a specific interest in identifying strengths frail older people with a higher QoL still have.

**Methods:**

Semi-structured interviews were held with community-dwelling, frail older people with higher (*n* = 16) and lower levels of QoL (*n* = 18). Frailty was assessed with the Comprehensive Frailty Assessment Instrument, which measures environmental, physical, psychological, and social frailty. Other quantitative measures included socio-demographic characteristics, overall QoL, meaning in life, and mastery. The qualitative part focused on the meaning and maintenance of QoL (among other factors), despite being frail. Possible explanations for discrepancies in QoL were explored.

**Results:**

Frail older people with a higher QoL were older, had lower levels of psychological frailty, and reported higher meaning in life compared to those with a lower QoL. Outcomes of qualitative analysis showed that participants in the high QoL subgroup adapted more effectively to difficulties, had more things in prospect, performed more activities, and were more satisfied with their social network compared to the low QoL subgroup.

**Conclusion:**

This exploratory study suggests possibilities to promote and improve QoL by strengthening specific resources among frail older people.

**Electronic supplementary material:**

The online version of this article (10.1186/s12877-017-0641-y) contains supplementary material, which is available to authorized users.

## Background

Although frailty is often described as merely a physical construct [1], there is a growing tendency to conceptualize frailty from a multidimensional perspective, where environmental, psychological, and social factors are taken into account as well [2]. Prevalence rates of multidimensional frailty vary between 4.2–59.1% across studies, depending on the definition and the population included [3]. Multidimensional frailty is associated with higher age [3, 4] and may lead to disability, hospitalization, early institutionalization, and death [5, 6]. Hence, it might threaten the wish most older people have to age in place [7]. Moreover, aging in place is stimulated from a policy perspective, for example to reduce the overall costs of institutionalization [8]. Early detection and prevention of frailty are important topics for research, policy, and clinical practice because of the growing number of people aged 65 years and over [9], the negative consequences of frailty, and the necessity of enabling aging in place.

Current approaches on frailty seem to be dominated by a “deficit approach.” They mainly focus on things people cannot do any longer and the risks of adverse outcomes [5, 6], or define frailty as an accumulation of deficits [10]. Despite this deficit approach in research, older people themselves seem less focused on shortages. A recent qualitative study showed that older people favor receiving support that improves their autonomy and well-being, instead of interventions which focus on diseases and dysfunctions [11, 12]. Besides, older people dislike an approach in which every older adult is perceived as someone with (a risk of) deficits [11]. Furthermore, qualitative studies revealed negative consequences of stereotyping. For example, when people were labeled as frail by others, this actually made them feel frail, and they behaved accordingly [13]. This suggests that a strengths-based approach, which offers the opportunity to get a better understanding of people’s strengths and abilities [14, 15], might be of value for frailty prevention strategies.

As frail older people still can have a good perceived quality of life (QoL), this might be an important entry for such a strengths-based approach. For example, previous research has shown that nearly 50% of the participants who were frail at least on the physical domain still reported a good to excellent QoL [16]. In another study, 46% of physically frail older women reported a good QoL [17], while in a qualitative study 8 out of 11 frail participants reported a satisfactory to good QoL [18]. Qualitative studies that have investigated what QoL means to (frail) older people revealed several important factors: social contacts, (physical) health, psychological well-being, being able to perform activities, and having enough facilities at home and in the neighborhood [18]. Nonetheless, little is known about factors that may actually contribute to a high QoL in frail older people. Although it has been found that frail older people with a higher age report higher levels of QoL [19], as well as those who compare themselves with people in a worse situation [18], the focus of these studies was not to identify strengths in frail older people. However, that is exactly what is important while aiming for a strengths-based approach.

The aim of this mixed-methods study was twofold: (1) to identify characteristics of community-dwelling, frail older people with higher and lower levels of QoL, respectively; and (2) to explain discrepancies in self-reported QoL, with a specific interest in identifying strengths that community-dwelling, frail older people with a higher QoL still have. Herewith, the focus was not on investigating associations between frailty and QoL, but on investigating which factors might explain differences in QoL among frail older people, as it seems particularly important to make (strength-based) interventions more tailored in this vulnerable population. While some studies assess multiple domains of QoL, this study focuses on overall QoL, which is defined as ‘an individual’s overall satisfaction with life and general sense of well-being’ [20], and for which one question seems to be a particular adequate measure [21].

## Methods

### Study design and participants

This study included a subsample of a larger mixed-methods study within the D-SCOPE project (Detection, Support and Care for Older people: Prevention and Empowerment, http://www.d-scope.be), which was conducted between November, 2015 and April, 2016. The D-SCOPE project aims at early detection and prevention of multidimensional frailty to enable ageing in place, focusing on deficits as well as strengths older people have. Participants needed to be 60 years and over, and living in the community. Exclusion criteria were as follows: hospitalization, institutionalization, and not being able to answer simple questions. In total, 121 community-dwelling people aged 60 years and over were recruited. Purposeful sampling was used to oversample community-dwelling older people at risk for frailty [4]. Healthcare organizations in West Flanders, Belgium, selected potential participants from their client database, based on risk profiles for frailty [4], and provided information about the study. For instance, they oversampled people aged 75 years and over, people without a partner, people with a migration background, and people who had moved in the past 10 years [4]. When older people approved to participate in the study, the healthcare organization provided the research team with their contact details. In addition, snowball sampling was used (i.e. participants contacted friends, family members and/or relatives and asked if they would like to participate. If yes: the participant provided the researcher with their contact details). The 121 older people gave their consent. The Ethics Commission in Humane Sciences of the Vrije Universiteit Brussel (Belgium) approved the study [reference ECHW_031].

For the current study, only frail older people with higher (≥ 8, *n* = 16) and lower levels of QoL (≤ 6, *n* = 18) were included. These cut-offs were based on tertiles to be able to compare sufficiently contrasting and nearly equally sized groups. “Frail” was defined as a combined score between 38.76 and 100 on the Comprehensive Frailty Assessment Instrument (CFAI, [22]) (explained in more detail under the heading Measures and data collection). The cut-off was based on a two-step cluster analysis that was conducted for each frailty domain, as well as for overall frailty. This analysis resulted in cut-offs for no-mild, mild, and high frailty (De Witte et al., available upon request). Six researchers (including author AvdV; all PhD students, 3 female) conducted the interviews, twice in the presence of an interpreter due to language barriers.

### Measures and data collection

A concurrent mixed-methods design was used. Both quantitative and qualitative data were assessed at once, in person, at the homes of the participants. First, the following socio-demographic characteristics were collected: age, gender, nationality, place of birth, marital status, and living arrangement. Next, overall cognition was measured in the D-SCOPE study using the Montreal Cognitive Assessment (MoCA) [23], which has been found to be a valid measure [24]. For the current study, MoCA scores were used to describe the overall sample as well as the high and low QoL subgroups. The MoCA assesses multiple cognitive domains, including short-term memory, visuospatial abilities (e.g., clock drawing), executive functioning, fluency, attention, concentration, working memory, language, and orientation. Third, multidimensional frailty was measured by administering the previously validated CFAI [2, 22], which comprises environmental (e.g., condition of the house), physical (e.g., walking difficulties), psychological (mood disorders and emotional loneliness), and social frailty (social loneliness and social support network). Fourth, participants were asked to rate their QoL (“On a scale from 0 to 10, how would you rate your QoL? By this, we mean how you feel about your life. Whether you are satisfied with your life and enjoy your life, for instance, and whether you find satisfaction in the life you live.”), meaning in life (“On a scale from 0 to 10, to what extent do you feel that your life is meaningful (e.g. worthwhile, purposeful, that you look forward to something or strive for something)?”), and mastery (“On a scale from 0 to 10, to what extent do you feel in control of what happens in your life?”) by means of Numeric Rating Scales ranging from 0 to 10, with lowest and highest scores as extreme values [25]. As we aimed to gain insight into overall QoL we asked for one general evaluation, which has been measured on a scale from 0 (lowest QoL) to 10 (highest QoL) in previous research as well [17]. For meaning in life and mastery, we aimed to measure the overall constructs as well. As we were dealing with a vulnerable population, one-item questions were used to measure these constructs. Similar approaches have been used previously [26, 27], though for comparability with the QoL meausure, we chose to measure these constructs on a scale from 0 to 10 as well.

The topic list of the qualitative interview included perceptions and experiences of the older people regarding the meaning of frailty, aspects of life that are important for QoL, the influence of frailty on QoL, factors that may help to maintain QoL despite being frail, positive and negative life events, and future perspective. An additional file shows an overview of the questions that were proposed during the qualitative survey [see Additional file 1]. The researchers received a 6-h theoretical and practical training on interview techniques in qualitative research. The interviews lasted between one and two-and-a-half hours, and were recorded with Audacity. Participants were informed that the aim of the interview was to gain insight into factors that enable older people to age in place. The interviews were fully transcribed, including words, speech particles, and expressions of feelings (e.g., laughter). Transcripts were not returned to participants for correction purposes.

### Data analysis

Initial analysis of quantitative and qualitative data was conducted independently. Regarding the quantitative data, descriptive statistics were calculated for socio-demographic characteristics, and MoCA and CFAI scores. To examine differences between the low (≤ 6) and high (≥ 8) QoL subgroups, Chi-square tests (categorical data; e.g. gender), and independent samples t-tests (scale data; age, MoCA, meaning in life, and multidimensional frailty) or Mann-Whitney U tests (QoL and mastery, due to non-normality of the data) were performed in SPSS Statistics for Windows, version 24.0 (Armonk, NY: IBM Corp).

**Fig. 1:**
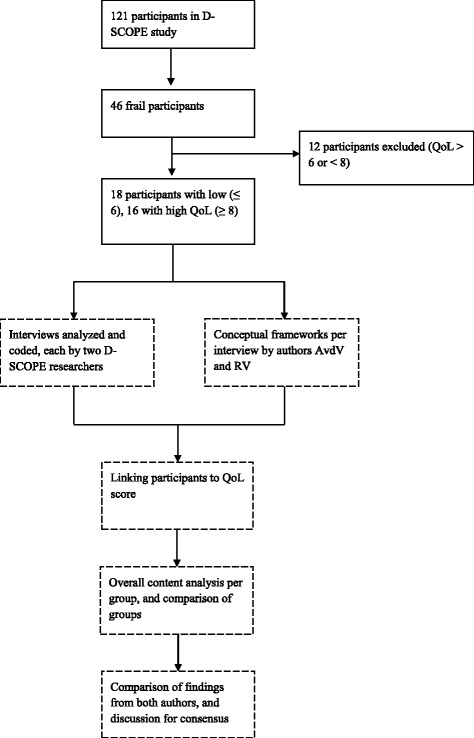
Flow chart of the selection process () and qualitative analysis procedure (----)

Regarding the qualitative data, the six researchers who conducted the interviews analyzed the data using MAXQDA (version 12 Standard, student license), based on the Qualitative Analysis Guide of Leuven [28]. To enhance the trustworthiness, each individual interview was analyzed and coded by two researchers independently. The two researchers were educated in different disciplines (e.g., educational sciences, gerontology, psychology, and nursing) to ensure that different perspectives were taken into account during the analyses. Most themes were derived in advance, matching the interview scheme. In addition, author AvdV read the full transcriptions of all 34 qualitative interviews and author RV independently read a random sample of five interviews from each QoL subgroup. Both authors were blinded for QoL scores. Summaries were made in order to obtain a conceptual framework per participant by (re) reading the interviews, trying to find the essential characteristics of each interview, and describing these findings conceptually [28]. Codes in MAXQDA as well as the conceptual frameworks were taken into account to perform an overall content analysis per subgroup. Thereafter, groups were compared. Finally, findings from both researchers (authors AvdV and RV) were compared and discussed to reach agreement. Fig. 1 displays the process of the qualitative analyses. A professional translator was consulted to translate quotes into English. Thereafter, the English quotes were compared with the original citations by author AvdV to ensure that the meaning of the quotes was preserved [29].

During the interpretation stage, findings from quantitative and qualitative results were compared, although emphasis was on the qualitative findings due to the exploratory design of the study.

## Results

### Sample characteristics

The mean age of the study sample was 80.7 years (range 66–94); 61.8% were female, 8.8% had a migration background, 61.8% were widowed, and 67.6% lived alone (Table 1).

**Table 1:** Characteristics of the sample, and comparisons per QoL subgroup

	Sample (*n* = 34)		Low QoL (*n* = 18)		High QoL (*n* = 16)		Group differences
Socio-demographics							
Age (mean) (SD)	80.7 (7.3)		**78.1 (6.5)**		**83.9 (7.0)** ^**a**^		*
Females (%) (n)	61.8	21	61.1	11	62.5	10	
Migration background (%) (n)	8.8	3	11.1	2	6.3	1	
Marital status (%) (n)							
Married	11.8	4	16.7	3	6.3	1	
Never married	11.8	4	5.6	1	18.8	3	
Divorced	14.7	5	27.8	5	–	0	
Widowed	61.8	21	50.0	9	75.0	12	
Living arrangement (%) (n)							
Alone	67.6	23	72.2	13	62.5	10	
With partner	11.8	4	16.7	3	6.3	1	
With child(ren)	17.6	6	11.1	2	25.0	4	
With others	2.9	1	–	0	6.3	1	
Cognition							
MoCA (mean, SD)	19.9 (4.1)		18.8 (4.4)^b^		21.2 (3.3)^c^		
Frailty (mean, SD)							
Overall	50.4 (8.6)		53.1 (8.3)		47.3 (8.3)		
Environmental	31.3 (17.7)		26.7 (19.2)		36.6 (14.7)		
Physical	81.3 (22.5)		82.0 (21.1)		80.5 (24.6)		
Psychological	40.0 (22.9)		**52.0 (16.2)**		**26.6 (19.7)**		***
Social	52.3 (18.6)		52.9 (15.1)		51.6 (22.4)		
Quality of life							
VAS-scale (median)	6		**6**		**9**		***
Meaning in life							
VAS-scale (mean, SD)	7.6 (1.9)		**6.9 (2.1)**		**8.4 (1.3)**		*
Mastery							
VAS-scale (median)	8		8		8.5		

*Note*: Low QoL = ≤ 6. High QoL = ≥ 8. *SD* Standard Deviation, *MoCA* Montreal Cognitive Assessment, *QoL* Quality of Life, *VAS* Visual Analogue Scale. Cognition (MoCA): range 0–30, cut-off for Mild Cognitive Impairment is ≤ 26. Frailty (Comprehensive Frailty Assessment Instrument): range 0–100 per domain, with higher scores indicating more severe levels of frailty

*p < .05. ***p < .001

^a^ *n* = 15. ^b^ *n* = 17. ^c^ *n* = 13

### Quantitative comparison

The outcomes of the quantitative analyses showed that participants in the high QoL subgroup (≥ 8; *n* = 16) were significantly older, had lower (less severe) levels of psychological frailty, and reported higher scores on meaning in life compared to the low QoL subgroup (≤ 6; *n* = 18) (Table 1).

### Qualitative comparison

The following five main themes were covered during the interviews: frailty, well-being, dealing with problems, life events, and future perspective.

#### Theme 1: Multidimensional frailty

The majority of participants in both subgroups did not identify themselves as frail. However, most reported physical health problems, and difficulties in at least one of the following domains: social relations, psychological functioning, the home environment, and cognition. Some examples of physical problems were loss of strength, mobility problems, and limited vision or hearing. Consequences of limited physical abilities were mentioned as well, such as losing confidence due to previous falls. A male participant (aged > 75) mentioned: *“I used to love going fishing. I had to get rid of everything. I couldn't see my float anymore.”* Regarding social relations, participants in both subgroups mentioned feelings of loneliness or losing social contacts, for example due to death or health problems of friends and family members. Concerning psychological functioning, participants felt more vulnerable, worried more often, and were more sensitive to things other people said compared to before. For example, a female participant (aged > 80) told: *“In the past, I would have said ‘hey, don't worry about it.’ But when you're old, you don't do that anymore. That’s strange, isn’t it? You just don’t have the strength anymore.”* Two participants reported a history of depression, and a male participant (aged > 75) said: “*Maybe, after you leave, I'll simply fall to bits this evening. Sometimes I get so sad all of a sudden that she’s no longer with me [late wife].”* Regarding their living environment, some participants needed adaptations (e.g., a shower instead of bathtub), or had difficulties walking the stairs. Negative aspects about the home environment were lack of shops in the neighborhood, not having as much contact with neighbors as preferred, new neighbors who were less likely to help, or losing interactions. For example: *“The first generation of people to live here were all friends. We’d go to concerts together, we did things with one another. The second generation, they were friendly folks, but they weren’t really friends like before. And now there are a few older people living here, who don’t get around as well as they used to, and who are pretty isolated too, actually. Plus a number of young people, a bunch of foreigners who don’t even say ‘good morning’ when you see them... time was, you wouldn’t dream of not doing that”* (woman, aged > 70). Some participants reported (being confronted with) memory problems, while others feared developing cognitive problems. In addition, trouble keeping up in today’s society was mentioned a couple of times in both subgroups.

A few discrepancies regarding multidimensional frailty between both QoL subgroups were observed. Some participants in the high QoL subgroup mentioned positive stories about their social relations. For example, a male participant (aged > 85) never felt lonely because he always had dinner together with his children. However, in contrast to the high QoL subgroup, participants in the low QoL subgroup mentioned a couple of times that they did not have enough financial resources to be able to perform desired activities.

#### Theme 2: Well-being

##### Subtheme 1: Quality of life

In both QoL subgroups, participants mentioned the following aspects as important for their QoL: activities (e.g., gardening); autonomy/independence; sufficient financial resources; (physical) health; optimism; pets; social contacts with their partner, family members, neighbors, and/or friends; and traveling.

By comparing both subgroups, it appeared that only participants in the high QoL subgroup mentioned that having something in prospect and being of value to and/or helping others was important for their QoL. For example: “*I have to say, no matter how low you’re feeling, when you do that [help others, ed.] and they give you that look... It just makes your whole day, doesn’t it? It’s amazing how that works”* (man, aged > 75). In contrast, in the low QoL subgroup, “unmet needs” were revealed. Participants mentioned feelings of loneliness, insufficient contact with family members, or inability to perform the activities they would like to do. In addition, a female participant (aged > 65) told: *“Um, to have a good quality of life and to live with purpose, it’s primarily important to have good health. So you need to be healthy, and to have financial resources as well. You need those, too—because when you can’t afford the things you want...,”* while she also said: *“That’s something else I find exhausting. I mean, because I’m constantly having to pinch pennies.”*

##### Subtheme 2: Meaning in life

Regarding meaning in life, the following was mentioned to be important in both subgroups: aging in place, family bonds, going out, pets, having social contacts, and being of meaning to others. For example, a male participant (aged > 85) told: *“If the children weren’t around any longer, I wouldn’t care a thing about living. Not a thing. Then I might as well just come right out and say I want to die, in that case. That’s something that I’m quite satisfied with, something that keeps me busy. Ah, yes, of course. Today I’m going to make soup.”*

When comparing both subgroups, having something to look forward to (e.g., seeing grandchildren grow up or going out with a friend each Saturday) was mainly mentioned in the high QoL subgroup. Once again, unmet needs were mentioned in the low QoL subgroup. For example, a female participant (aged > 65) told that her children and grandchildren, who were important to her meaning in life, lived abroad. As a consequence she had not much to look forward to.

#### Theme 3: Dealing with problems

##### Subtheme 1: Individual factors

Participants in both subgroups mentioned acceptance, active problem-solving, expressing emotions/feelings, optimism, religion, seeking distraction (e.g., performing hobbies) and comparing themselves with those in a worse situation as important factors to deal with problems. Examples of active problem-solving were memory training and remaining physically active despite limitations.

However, some strategies to deal with problems seemed more prevalent in the high QoL subgroup. For example, participants replaced activities (e.g., *“I used to still be able to go for bike rides, now I visit comrades, friends and family”*; man, aged > 80), and actively sought social support from friends and family members, which was less pronounced in the low QoL subgroup. In addition, participants with higher levels of QoL tried not to dwell in the past or on things they were no longer able to do. Instead, they focused on positive aspects (e.g., things they still could do), or tried to change the situation they were in. Furthermore, they seemed more convincing in how they dealt with problems. For example, a male participant (aged > 80) responded as follows to the question, *“And you tried to solve the problem?”: “I didn’t try to – that’s what I did.”* In contrast, strategies to deal with problems applied by participants with lower levels of QoL, such as optimism, did not always seem to be effective and long-lasting. For example, a female participant (aged > 80) responded to the question, *“What do you think an older person should do in order to preserve their quality of life?”* with: “*Stay optimistic. Keep being an optimist. ... That gets harder as you go on. Then you ask yourself: ‘What’s left for me?’ I’m pretty much left to my own devices ... Why am I still here?”* Additionally, passive reacting was mentioned in the low QoL subgroup, although this did not always seem to be effective. For example, a female participant (aged > 65) told: *“And that I’m coming here alone, too. And then the emptiness, the loneliness especially. And then I just watch TV, whatever’s on, and kind of zone out. And then it’s exactly like my brain has been numbed, just a little.”* Moreover, she passively waited until her friends called her, even during times when she needed social contact.

##### Subtheme 2: Neighborhood and living environment

In both subgroups, some participants who lived together with a child mentioned that this enabled them to age in place. Other positive aspects about the home environment were having a one-story house, receiving help from neighbors, living near all facilities, and feeling attached (e.g., a female participant (aged > 70) was emotionally attached to her house because her deceased husband had built everything). No noticeable discrepancies were observed when comparing the high and low QoL subgroup.

##### Subtheme 3: (In) formal care and social support

The majority of participants in both subgroups received informal care from family members, neighbors, and one participant even from her former son-in-law (woman, aged > 85). Most participants were positive about the help they received. Regarding formal care, participants mentioned the importance of social support (i.e., being able to tell someone about problems, and having a trustworthy relationship), and that it was reassuring to have someone to rely on, more than the actual care they received. A female participant (aged > 80) told: *“I’m happy whenever I see her. Because I see her as—I’d never say, ‘the hired help.’ No, no—I always say ‘my friend.’”* Negative aspects about receiving help included being confronted with things they could not do any longer, as well as (the fear of) becoming dependent: *“I’d really like to be able to dine out from time to time. But I can’t do that by myself. I always have to have someone else go with me. I went [to a restaurant] once with the carer, but then she has to ask for permission from her, uh, company and also, well, I can’t pay for her meal all the time. So you see, that’s gone too.”* (woman, aged > 85). In addition, some participants preferred to arrange things themselves. For instance, a male participant (aged > 80) stated: *“We do everything ourselves, because I was sick of it. No, no, no... The children think they can do a better job than we can. No, we can take care of it on our own.”* Lastly, some negative experiences with formal care were mentioned, such as feeling treated like a number.

Some discrepancies appeared between both subgroups. In the high QoL subgroup, more participants used assisting aids compared to the low QoL subgroup, and this actually enabled them to do the things they wanted. For example, a female participant (aged > 85) told: *“And when the weather’s fair I can walk to my fence and back 10 times in an afternoon, with my little rolly-car [rollator], and that makes me feel good. Someone’s bound to walk by so you can have a bit of chit chat.”* In addition, participants in the high QoL subgroup were more involved in activities offered by welfare organizations, which gave them something to look forward to. Regarding social support, positive stories were mentioned more often in the high QoL subgroup compared to the low QoL subgroup. For example, having social contacts with and feeling supported by others really helped participants, and provided comfort: *“Sometimes just getting a hold of someone by phone, or that I just finished talking with somebody and I think, ‘So, I feel worlds better’.”* (woman, aged > 75). Although less often, a few participants in the low QoL subgroup explicitly mentioned positive aspects about social contacts as well: *“That’s a real comfort to me, getting out of the house and sitting at a table with those guys and drinking coffee and such.”* (man, aged > 80).

#### Theme 4: Life events

Participants in both subgroups mainly experienced negative life events, such as changes in their (physical) health, losses of people around them, or troubles within their families. For example, a female participant (aged > 85) recently lost her youngest brother, who was like a son to her because she raised him. However, some positive life events were mentioned as well, such as an improvement of health after stopping smoking and the birth of great-grandchildren: *“It feels as though I’m stronger and healthier, like I used to be, because of those children [birth of grandchildren]. Like I won the lottery”* (woman, aged > 85). In the low QoL subgroup, changes in living situation were mentioned, which were positive in most cases, sometimes due to a higher comfort level. However, the house of a female participant (aged > 80) burnt down, which caused her to lose tangible memories: “*And then the house having burnt down. That’s my lot in life, I can’t do anything to fix that misfortune. Those are all my memories and what have you, and they’re all gone. And yes... I still miss that. That’s over now. It can’t be brought back. No. I fully understand that.”* Lastly, a female participant (aged > 75) in the low QoL subgroup recently lost her job due to illness, and as a consequence also lost social contacts.

No clear discrepancies were observed between the two subgroups.

#### Theme 5: Future perspective

Some participants still had really specific dreams, such as going out for dinner once again, winning the lottery, swimming with dolphins, remaining independent, or going back to their place of birth.

When comparing both subgroups, it appeared that wishes regarding the future were more often pronounced in the high QoL subgroup. In contrast, in the low QoL subgroup it was more common that participants did not dare to dream or did not have any expectations, and they said things like: “*What am I still doing here?”* (man, aged > 85), or even feared the future. In response to the question, “*Do you think your life might change within a year?”* a male participant (aged > 80) replied, *“I hope not, but I worry it will. I might get more of the shakes [*i.e.*, Parkinson’s disease].”*

## Discussion

This mixed-methods study is the first aimed at identifying discrepancies between community-dwelling, frail older people with higher and lower levels of self-reported, overall QoL, focusing on potential strengths frail older people with higher levels of QoL still have. Despite similarities regarding perceived frailty or vulnerabilities according to the qualitative data, the quantitative data showed that people in the high QoL subgroup had lower levels of psychological frailty. In addition, higher age, higher levels of meaning in life (both quantitative data), having things in prospect, being of value/meaning to others, being able to cope with or adapt to difficulties, performing activities, and satisfaction with social network appeared to be factors that can distinguish frail older people with a higher QoL from those with a lower QoL. In addition, in the low QoL subgroup more unmet needs were experienced regarding factors important for QoL and meaning in life.

Participants in the high QoL subgroup seemed to have more effective ways of coping with and/or adapting to difficulties. For example, they focused on things they still were able to do, or replaced activities they were no longer able to perform. Indeed, previous research has shown that being able to cope with difficulties was important for life satisfaction in people aged 80 years and over who were assessed during and after rehabilitation [30]. In addition, it has been found that disabled persons who were able to adapt reported higher levels of QoL compared to those who did not [31], which is in line with our findings. Furthermore, both our quantitative and qualitative findings indicate that meaning in life is associated with QoL in frailty, along with having things in prospect. Related to this, previous research has shown that meaning in life contributes to QoL in people with chronic diseases [32], while not having a purpose in life has been found to be associated with lower levels of QoL [31]. Nonetheless, being able to cope and meaning in life have been found to be associated as well [33]. Therefore, it could be argued that meaning in life is a part of the relationship between coping and QoL.

Although no clear differences were found in the qualitative interviews regarding the experienced level of frailty, while exploring the other quantitative findings, lower levels of psychological frailty were observed in the high QoL subgroup. Indeed, previous research has shown that feeling down, which is an important aspect of psychological frailty, is associated with lower levels of environmental, social, physical, and psychological QoL [34]. In addition, psychological frailty has been found to predict past, present, and future activities [35]. This could be related to our qualitative finding that frail older people with a higher QoL performed activities more often, while this was mentioned to be important for QoL in both subgroups. With regard to the finding that age might play an important role, previous research has found that being older was associated with a higher QoL in frailty as well [19]. They argued that this could be due to the fact that older people had been able to adapt to their frailty. However, for the current study it is unknown whether participants in the high QoL subgroup indeed had been frail for a longer period of time than participants in the low QoL subgroup. Nonetheless, being able to adapt (e.g., by replacing activities one was no longer able to do) has been found to be important for higher levels of QoL.

Regarding QoL itself, previous research has already shown that psychological well-being, social contacts, and being able to perform activities are important aspects of QoL in older people [18]. Indeed, these factors were mentioned to be important for QoL in this study as well. However, our findings add that these factors might actually be related to higher levels of QoL in frailty, as more unmet needs were mentioned in the low QoL subgroup. Whereas previous research has already shown that unmet needs are associated with lower levels of QoL [36], this study identified specific unmet needs in frail older people with a lower QoL. For example, not having enough financial resources was only mentioned in the low QoL subgroup. While previous research has shown that financial resources had no significant effect on the association between frailty and well-being [37], they have not investigated whether income actually fitted the needs of the older people, while this seems to be what is important according to our qualitative findings.

An unexpected finding might be that the majority of participants in both subgroups received (in) formal care as previous research has shown that support from informal caregivers enabled frail older people to maintain their well-being [38]. We therefore would expect to see lower levels of (in) formal care in the low QoL subgroup. However, although both subgroups mentioned the social aspect to be important rather than the actual care they received from (in) formal caregivers, the low QoL subgroup less often pronounced that they actually felt supported by their social network, while this was one of the important aspects for higher levels of well-being in the previous study [38].

### Strengths and limitations

This study has several strengths. First of all, we adopted a mixed-methods design, and with the qualitative part we were able to explore experiences of frail older people more in-depth, taking a lot of different individual perspectives into account. Second, to reduce the risk of bias in personal interpretation, all interviews were coded independently by two researchers with different educational backgrounds, and group analyses were performed independently by two researchers as well. While author AvdV was involved in conducting the interviews and analyzing them in the first stage, author RV was an independent researcher who did not conduct any interviews nor was involved in the research project, to enable the trustworthiness of the findings. Lastly, by providing quotes in the results section, readers are enabled to interpret findings themselves.

However, this study has several limitations as well. First, the small sample size might have influenced quantitative findings, as the statistical power was relatively low. Second, only five participants in the low QoL subgroup (≤ 6) scored 5 or lower on QoL, and none below 4, whereas five participants in the high QoL subgroup (≥ 8) scored a 10. Therefore, the contrast between subgroups might be not as large as needed to distinguish substantial differences in people with lower and higher QoL levels. Third, QoL might fluctuate from day to day [39], which was mentioned by one of the participants in the high QoL subgroup as well, but no repeated measures were conducted. Fourth, by assessing overall QoL with one overarching question, it could be argued that participants had different operationalization’s in mind [40]. Nonetheless, overall QoL can be defined as ‘an individual’s overall satisfaction with life, and one’s general sense of personal wellbeing’ [20, 21]. Fifth, informal caregivers were present during three interviews in the high QoL subgroup and during one interview in the low QoL subgroup, which might have influenced participants’ responses as they might have given socially desirable responses. Linked to this, an interpreter joined the interview in the case of language barriers (once in both subgroups), which might have affected the results as well [41]. In addition, generalizability of findings is limited as only frail older people were included, although the frailest might have refused to take part. Lastly, participants in both subgroups had relatively low scores on the MoCA. However, a systematic review showed that in population-based cohorts many participants score below the cut-off of 26 for MCI, and it is argued that the threshold should be lower [42]. In addition, we intended to include people at risk for frailty, which might explain these cognitive vulnerabilities.

## Conclusion

Frail older people with a higher QoL seem to have better and more effective ways to cope with and/or adapt to difficulties. In addition, they report higher levels of meaning in life, seem to have more things in prospect, are older, have lower levels of psychological frailty, perform more activities, and are more satisfied with their social relationships compared to frail older people with a lower QoL. On the contrary, frail older people with lower levels of QoL report more unmet needs regarding their QoL and meaning in life.

### Implications for future research

While this study aimed to identify individual perspectives and therefore was explorative, by means of a quantitative study approach it will be possible to examine whether for example coping strategies, having something in prospect, and meaning in life actually contribute to QoL in frailty on a larger scale. Hereby, a longitudinal study with repeated measures might be needed as QoL might change over time. Furthermore, future research should explicitly ask whether or not participants feel frail. Previous research has shown that older people who were classified as frail do not always perceive themselves as frail [43], and “experienced frailty” may influence their self-reported QoL as well.

### Implications for clinical practice

While current clinical practice in frailty mainly focuses on the prevention of negative outcomes, such as delaying functional decline [44] or institutionalization [45], results from this study suggest possibilities to promote and improve QoL by strengthening specific resources among frail older people. As older people indicate that they wish to focus on things they still can do [11], such a strengths-based approach seems to be a promising way to work more preventatively. Therewith, clinical practice should focus on improving ways older people adapt to or cope with problems, psychological well-being, improving their meaning in life, making sure that people have something in prospect, and social contacts, as these factors contribute to QoL even in frailty. It is expected that this will contribute to aging in place with a good QoL.

## Additional file

Additional file 1: Overview quantitative and qualitative survey. (PDF 319 kb)

## Abbreviations

CFAI: Comprehensive Frailty Assessment Instrument
MoCA: Montreal Cognitive Assessment
QoL: Quality of life

## References

[1] Fried LP, Tangen CM, Walston J, Newman AB, Hirsch C, Gottdiener J (2001). Frailty in older adults: evidence for a phenotype. J Gerontol A Biol Sci Med Sci.

[2] De Witte N, Gobbens R, De Donder L, Dury S, Buffel T, Schols J.M.G.A, Verté D. The comprehensive frailty assessment instrument: development, validity and reliability. Geriatr Nurs 2013;34(4):274–281. 10.1016/j.gerinurse.2013.03.002 23608069

[3] Collard RM, Boter H, Schoevers RA, Oude Voshaar RC (2012). Prevalence of frailty in community-dwelling older persons: a systematic review. J Am Geriatr Soc.

[4] Dury S, De Roeck E, Duppen D, Fret B, Hoeyberghs L, Lambotte D, et al. Identifying frailty risk profiles of home-dwelling older people: focus on sociodemographic and socioeconomic characteristics. Aging Ment Health. 2016; 10.1080/13607863.2016.1193120. 10.1080/13607863.2016.1193120 27267783

[5] Gobbens RJJ, van Assen MALM, Luijkx KG, Schols JMGA (2012). The predictive validity of the Tilburg frailty indicator: disability, health care utilization, and quality of life in a population at risk. Gerontologist.

[6] Song X, Mitnitski A, Rockwood K (2010). Prevalence and 10-year outcomes of frailty in older adults in relation to deficit accumulation. J Am Geriatr Soc.

[7] Wiles JL, Leibing A, Guberman N, Reeve J, Allen RES (2012). The meaning of “aging in place” to older people. Gerontologist.

[8] Scharlach A (2011). Creating aging-friendly communities in the United States. Ageing Int.

[9] European Commission (2014). The 2015 aging report.

[10] Rockwood K, Mitnitski A (2007). Frailty in relation to the accumulation of deficits. J Gerontol A Biol Sci Med Sci.

[11] Lette M, Baan CA, van den Berg M, de Bruin SR (2015). Initiatives on early detection and intervention to proactively identify health and social problems in older people: experiences from the Netherlands. BMC Geriatr.

[12] van Kempen JAL, Robben SHM, Zuidema SU, Olde Rikkert MGM, Melis RJF, Schers HJ (2012). Home visits for frail older people: a qualitative study on the needs and preferences of frail older people and their informal caregivers. Br J Gen Pract.

[13] Warmoth K, Lang IA, Phoenix C, Abraham C, Andrew MK, Hubbard RE, Tarrant M (2015). “Thinking you're old and frail”: a qualitative study of frailty in older adults. Ageing Soc.

[14] Graybeal C (2001). Strengths-based social work assessment: transforming the dominant paradigm. Fam Soc.

[15] Minimol K (2016). Risk assessment and strengths based case Management in Elderly Care–Scope of social work practice. Artha-J Soc Sci.

[16] Ament BHL, de Vugt ME, Verhey FRJ, Kempen GIJM (2014). Are physically frail older persons more at risk of adverse outcomes if they also suffer from cognitive, social, and psychological frailty?. Eur J Ageing..

[17] Zaslavsky O, Woods NF, LaCroix AZ, Cauley JA, Johnson KC, Cochrane BB, Sagi SZ (2016). Identification of risk factors for mortality and poor-quality-of-life survival in frail older women participating in the Women's Health Initiative observational study. J Am Geriatr Soc.

[18] Puts MTE, Shekary N, Widdershoven G, Heldens J, Lips P, Deeg DJH (2007). What does quality of life mean to older frail and non-frail community-dwelling adults in the Netherlands?. Qual Life Res.

[19] Bilotta C, Bowling A, Casè A, Nicolini P, Mauri S, Castelli M, Vergani C (2010). Dimensions and correlates of quality of life according to frailty status: a cross-sectional study on community-dwelling older adults referred to an outpatient geriatric service in Italy. Health Qual Life Outcomes.

[20] Spilker B, Revicki DA, Spilker B (1996). Taxonomy of quality of life. Quality of life and Pharmacoeconomics in clinical trials.

[21] Arnold R, Ranchor AV, Sanderman R, GIJM K, Ormel J, TPBM S (2004). The relative contribution of domains of quality of life to overall quality of life for different chronic diseases. Qual Life Res.

[22] De Witte N, Gobbens R, De Donder L, Dury S, Buffel T, Verté D, Schols JMGA. Validation of the comprehensive frailty assessment instrument against the Tilburg frailty indicator. Eur Geriatr Med. 2013;4(4):248–54. 10.1016/j.eurger.2013.03.001. 10.1016/j.gerinurse.2013.03.002 23608069

[23] Nasreddine ZS, Phillips NA, Bédirian V, Charbonneau S, Whitehead V, Collin I, Cummings JL, Chertkow H (2005). The Montreal cognitive assessment, MoCA: a brief screening tool for mild cognitive impairment. J Am Geriatr Soc.

[24] Lam B, Middleton LE, Masellis M, Stuss DT, Harry RD, Kiss A, Black SE (2013). Criterion and convergent validity of the Montreal cognitive assessment with screening and standardized neuropsychological testing. J Am Geriatr Soc.

[25] Polit DF, Beck CT (2008). Nursing research. Generating and assessing evidence for nursing practice.

[26] Takkinen S, Ruoppila I (2001). Meaning in life as an important component of functioning in old age. I nt J Aging Hum Dev.

[27] Borneman T, Ferrell B, Puchalski CM (2010). Evaluation of the FICA tool for spiritual assessment. J Pain Symptom Manag.

[28] Dierckx de Casterlé B, Gastmans C, Bryon E, Denier Y (2012). QUAGOL: A guide for qualitative data analysis. Int J Nurs Stud.

[29] van Nes F, Abma T, Jonsson H, Deeg D (2010). Language differences in qualitative research: is meaning lost in translation?. Eur J Ageing.

[30] Aberg AC, Sidenvall B, Hepworth M, O’Reilly K, Lithell H (2005). On loss of activity and independence, adaptation improves life satisfaction in old age–a qualitative study of patients’ perceptions. Qual Life Res.

[31] Albrecht GL, Devlieger PJ (1999). The disability paradox: high quality of life against all odds. Soc Sci Med.

[32] Bernard M, Braunschweig G, Fegg MJ, Borasio GD, et al. Meaning in life and perceived quality of life in Switzerland: results of a representative survey in the German, French and Italian regions. Health Qual Life Outcomes. 2015;13:160.10.1186/s12955-015-0353-y. 10.1186/s12955-015-0353-y PMC4587717 26416234

[33] Park CL, Malone MR, Suresh DP, Bliss D, Rosen RI (2008). Coping, meaning in life, and quality of life in congestive heart failure patients. Qual Life Res.

[34] Gobbens RJJ, Luijkx KG, van Assen MALM (2013). Explaining quality of life of older people in the Netherlands using a multidimensional assessment of frailty. Qual Life Res.

[35] Coelho T, Paúl C, Fernandes L (2015). Physical, psychological and social frailty in prediction of disability and quality of life. Eur Psychiatry.

[36] Hansen DG, Larsen PV, Holm LV, Rottmann N, Bergholdt SH, Søndergaard J (2013). Association between unmet needs and quality of life of cancer patients: a population-based study. Acta Oncol.

[37] Hubbard RE, Goodwin VA, Llewellyn DJ, Warmoth K, Lang IA (2014). Frailty, financial resources and subjective well-being in later life. Arch Gerontol Geriatr.

[38] Lloyd A, Kendall M, Starr JM, Murray SA (2016). Physical, social, psychological and existential trajectories of loss and adaptation towards the end of life for older people living with frailty: a serial interview study. BMC Geriatr.

[39] Moons P, Budts W, De Geest S (2006). Critique on the conceptualisation of quality of life: a review and evaluation of different conceptual approaches. Int J Nurs Stud.

[40] Rogerson RJ (1995). Environmental and health-related quality of life: conceptual and methodological similarities. Soc Sci Med.

[41] Squires A (2009). Methodological challenges in cross-language qualitative research: a research review. Int J Nurs Stud.

[42] Davis DHJ, Creavin ST, Yip JLY, Noel-Storr AH, Brayne C, Cullum S. Montreal cognitive assessment for the diagnosis of Alzheimer’s disease and other dementias (review). Cochrane Database Syst Rev. 2015;10. 10.1002/14651858.CD010775.pub2 10.1002/14651858.CD010775.pub2 PMC6682492 26513331

[43] Puts MTE, Shekary N, Widdershoven G, Heldens J, Deeg DJH (2009). The meaning of frailty according to Dutch older frail and non-frail persons. J Aging Stud.

[44] Ruikes FGH, Zuidema SU, Akkermans RP, Assendelft WJJ, Schers HJ, Koopmans RTCM (2016). Multicomponent program to reduce functional decline in frail elderly people: a cluster controlled trial. J Am Board Fam Med.

[45] de Almeida MJ, Declercq A, Cès S, Van Durme T, Van Audenhove C, Macq J. Exploring home care interventions for frail older people in Belgium: a comparative effectiveness study. J Am Geriatr Soc. 2016;64(11):2251–6. 10.1111/jgs.14410 27676585

