# Supplementary material for: Explaining discrepancies in self-reported quality of life in frail older people: a mixed-methods study
Source: BMC Geriatr. 2017 Oct 26;17:251. doi: 10.1186/s12877-017-0641-y (PMC5659025; doi:10.1186/s12877-017-0641-y)
Supplement: Additional file 1: — Overview quantitative and qualitative survey. (PDF 319 kb) [file 12877_2017_641_MOESM1_ESM.pdf]

Overview quantitative and qualitative survey

Table 1. Overview quantitative survey

**Domains**	**Specific variables / measurement instrument**	**Description**
Socio-demographic characteristics		
	Age	
	Gender	
	Nationality	
	Country of birth	
	Marital status	
	Living arrangement	
Cognition		
	Montreal Cognitive Assessment (MoCA) (Nasreddine et al., 2005)	Multiple cognitive domains, including, amongst others, short-term memory, executive functioning, and orientation
Frailty		
	Comprehensive Frailty Assessment Instrument (CFAI; De Witte et al., 2013)	Environmental, physical, psychological, and social frailty
Quality of life		1-item, 10-point scale
Meaning in life		1-item, 10-point scale
Mastery		1-item, 10-point scale

Table 2. Overview semi-structured qualitative survey

**Topics**	**Questions**
Frailty	▪ It is often said that people become frail as they age. How do you experience this yourself?<br>• What does frailty mean to you?<br>• Do you feel more or less frail than other people your age? *(Do they feel frail?)*
Quality of life, meaning in life	▪ Do you believe that frailty affects the quality of life and meaning of life?<br>• What is your view on that? Do you experience that yourself?<br>• What are things that contribute to your quality of life, despite being frail?<br>• What makes your life meaningful (worthwhile, purposeful)?<br>• As a frail older person, what do you need to be able to lead a quality, meaningful life?
Mastery	▪ Do you feel like you have control over the things that happen in your life?

	• To what extent do you feel able to make your own decisions regarding what happens in your life?<br>• How can you keep control of your own life? What does that require?
Dealing with problems	▪ What should an older person do to maintain his/her quality of life / mastery / meaning in life when becoming frail?<br>• What is your own experience in that respect? How do you see that, in your own case?<br>• What factors influence this, do you think? (*Individual aspects, what part do (in)formal caregivers, the environment/neighborhood, … play?)*
Life-events	▪ Looking back on the last year, have there been any significant changes in your life?<br>• Can you describe important positive and negative changes that have happened in the past year?<br>• What was your most positive experience of the last year?<br>• In contrast to the high, did you perhaps also experience a low point during the last year?
Future perspective	▪ Do you expect your life to have changed in a year's time?<br>▪ Do you have any specific expectations for the future?<br>• What dreams do you have? Are there dreams you want to accomplish?<br>• What obstacles do you expect?<br>*(Do they think that they will have a quality life? That they will become frail?)*
